# Vitiligo: analysis of grafting versus curettage alone, using melanocyte morphology and reverse transcriptase polymerase chain reaction for tyrosinase mRNA

**DOI:** 10.1590/S1516-31802005000400006

**Published:** 2005-07-07

**Authors:** Carlos D'Aparecida dos Santos Machado, Fernando Augusto Almeida, Rodrigo Sestito Proto, Gilles Landman

**Keywords:** Vitiligo, Transplants, Tyrosinase, Polymerase chain reaction, Pathology, Vitiligo, Transplantes, Tirosinase, Reação em cadeia de polimerase, Patologia

## Abstract

**CONTEXT AND OBJECTIVE:**

Recent studies have indicated that vitiligo areas contain inactive or dormant melanocytes. Melanin synthesis is related to tyrosinase presence and indicative of active metabolic state. The aim of this study was to compare repigmentation, epidermal melanocyte distribution and tyrosinase mRNA detection through reverse transcriptase polymerase chain reaction, in tissue samples of vitiligo, before and after curettage, with or without subsequent autologous skin graft using a new method.

**DESIGN AND SETTING:**

Prospective, in the Department of Dermatology, Faculdade de Medicina do ABC, Santo André.

**METHODS:**

Two vitiligo areas were curetted. One subsequently received grafted normal sacral autologous skin, whereas the other had no further treatment. The curetted areas were examined after 30 days, to evaluate the degree of repigmentation. The melanocyte percentages and tyrosinase mRNA presence in normal skin and vitiligo areas, before and after curettage and grafting, were compared.

**RESULTS:**

Complete repigmentation was seen in all grafted areas, whereas non-grafted curetted vitiligo presented partial repigmentation. The melanocyte percentage in grafted areas was greater than in non-treated vitiligo skin (p = 0.01) and skin with curettage alone (p = 0.015). Tyrosinase mRNA was negative in 93.75% of non-treated vitiligo areas. After treatment (curettage alone or curettage and grafting), all lesions became positive for tyrosinase mRNA.

**CONCLUSION:**

Metabolically inactive or dormant melanocytes are probably present within vitiligo areas, and may be activated by exogenous or endogenous stimuli.

## RESUMO

**CONTEXTO E OBJETIVO:**

Estudos recentes têm indicado que áreas de vitiligo contêm melanócitos inativos ou dormentes. A síntese de melanina está relacionada com a presença de tirosinase é indicadora de estado metabólico ativo. O escopo deste artigo é comparar repigmentação, distribuição epidérmica de melanócitos e detecção do RNAm de tirosinase através da reação de polimerase em cadeia por transcriptase reversa, em amostras de tecido de vitiligo, antes e após curetagem, seguido ou não por uma nova técnica de enxerto autólogo de pele.

**TIPO DE ESTUDO E LOCAL:**

Prospectivo, na Disciplina de Dermatologia da Faculdade de Medicina do ABC, Santo André.

**MÉTODOS:**

Duas áreas de vitiligo foram curetadas. Uma foi subseqüentemente enxertada com pele autóloga sacral normal, enquanto a outra não recebeu nenhum enxerto. As áreas curetadas foram examinadas após 30 dias, para avaliar o grau de repigmentação. A porcentagem de melanócitos e a presença de RNAm de tirosinase de pele normal e de pele de vitiligo (antes e após curetagem apenas e antes e após curetagem seguida de enxertia) foram comparadas.

**RESULTADOS:**

Repigmentação completa foi observada em todas as áreas enxertadas, enquanto as áreas de vitiligo apenas curetadas apresentaram repigmentação parcial. A porcentagem de melanócitos nas áreas enxertadas excederam as áreas de vitiligo não tratados (p = 0.01) e as áreas tratadas com curetagem simples (p = 0,015). RNAm de tirosinase foi negativo em 93.75% das áreas não tratadas de vitiligo. Após tratamento (somente curetagem ou curetagem seguida de enxerto), todas as lesões tornaram-se positivas para RNAm de tirosinase.

**CONCLUSÃO:**

Melanócitos metabolicamente inativos ou dormentes estão provavelmente presentes dentro das áreas de vitiligo, e podem ser ativados por estímulos endógenos ou exógenos.

## INTRODUCTION

Vitiligo, a cutaneous achromia, is poorly understood and is related to three hypothetical pathogenetic mechanisms: **autotoxicity** (excessive endogenous production of phenol radicals, derived from dopaquinone oxidation into melanin products),^1^ **neurological** (damage of end-terminal portions of autonomic nerves)^2^ and **immunological** (anti-tyrosinase and TRP-2 autoantibodies, as well as through activation of cellular immunity).^3,4^

Although the end result of the disease is epidermal depigmentation, the presence or absence of remaining melanocytes within vitiligo areas, and also the possible melanocyte metabolic arrest are still controversial.^5-6^ Characterization of the melanocyte metabolic status is an essential matter. For many years, the dopa reaction for tyrosinase was the gold standard for determining melanocyte activity, just as the detection of melanosomes via electron microscopy was a condition for characterizing cells as melanocytes. These techniques are, however, unable to distinguish immature or metabolically inactive melanocytes. Recently, Le Poole^7^ (1993) developed new antibodies against melanoblasts (metabolically active cells) on the migration pathway from the neural crest to the skin, named NKI/beteb and A4 F11. Vitiligo lesions that gave a negative reaction to these antibodies were interpreted as being free of melanocytes. However, inactive melanocytes would not have tyrosinase activity and would therefore also result in negative reactions.

After hair pulling, germinative buds of the initial anagen phase of new hair growth express tyrosinase mRNA, which can be detected by means of the reverse transcriptase polymerase chain reaction (RT-PCR), whereas telogen phase hair follicles do not. This indicates that melanocytes may alternate cycles of activity with metabolic rest.^8^ Tyrosinase mRNA via RT-PCR has recently been used to detect melanoma micrometastasis.^9^ This technique has rarely been used to study melanocyte activity and melanogenesis, or to evaluate curettage and/or melanocyte skin graft for vitiligo.

There are many types of vitiligo treatment, including: melanogenesis stimula- tors^10-15^ immunomodulators^16-19^ depigmen- tating agents^20^ monoaminoxidase inhibi- tors^16^ and grafting.^21-24^

We decided to further investigate the melanocyte distribution in normal skin and vitiligo lesions, so as to establish the influence of skin graft and trauma on melanocyte metabolism.

## OBJECTIVE

To establish an autologous grafting protocol for longstanding vitiligo, in order to evaluate repigmentation and melanocyte distribution and the presence of tyrosinase mRNA, seen via RT-PCR, comparing curettage alone with curettage followed by normal skin graft.

## METHODS

### Patients

Between 1998 and 2000, 40 patients with vitiligo were studied (31 female and 9 male), with ages ranging from 11 to 71 years old. The types of vitiligo lesions presented were generalized (22 patients), segmental (16 patients) and perinevic (2 patients).

### Inclusion criteria

There had to have been no treatment that influenced melanogenesis over the preceding three months for the patient to be included in this study. Longstanding generalized vitiligo lesions needed to have been stable for at least one year.^25^ In order to avoid melanocytic contamination during the experiments, macules of area greater than or equal to 4 cm^2^ were chosen. The donor skin came from the sacral area. All patients agreed to the procedure and signed an informed consent form.

### Study groups

*Grafted group*: After curettage, one vitiligo lesion received a graft of normal skin, as described below. *Control group*: On the same patient, another lesion was only curetted, as a control for the grafted group.

### Graft harvest technique

**Figure 1:**
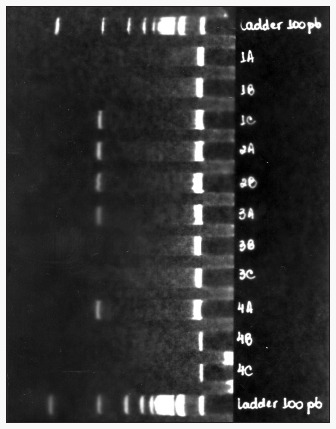
Normal skin graft procedure in curetted vitiligo area (marked inner square area).

After 2% lidocaine admixed with 1:50,000 adrenaline anesthesia had been administered, a 3 cm^2^ donor area of normal skin was scraped using a disposable 3 mm dermatological curette until punctate bleeding appeared. The tissue obtained was minced on a Petri dish and diluted in saline solution, until a soft paste was obtained (Figure 1). In the center of an area of vitiligo to be grafted, a 1 cm^2^ area was carefully scraped using a disposable 2 mm curette, to avoid touching the adjacent normal skin area, thereby preventing contamination with melanocytes. Normal skin was then placed on the scraped area of the skin. The control area was prepared in the same way, except that no normal skin was grafted onto the scraped area. The grafted and control areas were covered with a semi-permeable occlusive and adherent membrane, and gauze and adhesive tape were placed on top of this for seven days. The donor area was left open for second intention healing. After seven days, the dressings were removed and the wounds were examined and left without protection thereafter.

### Evaluation of clinical results

The wounds were examined after 7, 30, 60 and 180 days, in order to evaluate graft integration in the receptor area and the clinical signs of repigmentation (graded as absent, mild, moderate and intense), and also to evaluate the donor area recovery.

### Tissue harvesting and melanocyte counting

Skin tissue samples were obtained before grafting from the donor, receptor and control areas. Punch biopsies were performed on ten randomly chosen patients. Thirty days later, the receptor and control areas were again harvested. The specimens were fixed in 10% buffered formalin, embedded in paraffin and sectioned at thicknesses of 3 to 4 micrometers. The sections were stained with hematoxylin and eosin, and by the Fontana-Masson silver stain method. Two observers (CDSMF and GL) counted all cells of the epidermal basal layer, thus establishing a melanocyte versus keratinocyte ratio. The results were expressed as the mean melanocyte percentage of the total number of cells in the basal cell layer. The silver stain section was examined in order to determine which lesions had melanin granules on the epidermis.

### Tyrosinase mRNA by RT-PCR

Vitiligo tissue samples were obtained from sixteen randomly chosen patients as follows: a total of 24 biopsies from 12 receptor areas (before and 30 days after curettage), 40 biopsies from 20 control areas (before and 30 after days grafting), and 11 donor areas (before graft harvesting). In order to avoid melanocytic contamination, each biopsy was performed using a different punch device. Tissue samples were transported in 10 ml HAM-F10 (Gibco BRL, Life Technology, Gaitersburg, USA). Tyrosinase mRNA detection by means of RT-PCR was performed according to the previously described technique.^26^

### Statistical analysis

Wilcoxon tests were used in order to compare mean melanocyte percentages. P values < 0.05 were considered significant.

## RESULTS

### Pigmentation

**Figure 2:**
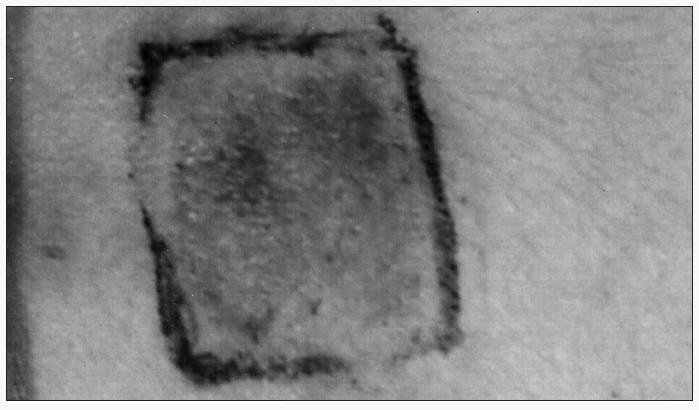
Hyperpigmentation, 40 days after normal graft procedure in vitiligo area.

After seven days, the curetted areas showed epidermal recovery, although erythema persisted for 30 days. Pigmentation was incipient among grafted patients and absent in the control group. Forty to 60 days after grafting, a moderate to intense degree of pigmentation and even hyperpigmentation was seen (Figure 2), while the control group had only a mild degree of pigmentation. After 180 days, this control group had a predominantly perifollicular pigmentation pattern.

Intense pigmentation was found in 16 grafted patients (40%).

After 30 days, six patients showed distant repigmentation of untreated vitiligo lesions (inverted isomorphism).

### Melanocyte count

Receptor areas had higher melanocyte percentages after grafting than in biopsies taken before the procedure (p = 0.01). The melanocyte percentages in grafted areas were also higher than in non-grafted curetted vitiligo skin (p = 0.015). Compared with the donor areas, control areas exhibited higher melanocyte percentages (p = 0.02). The melanocyte percentages in receptor areas before grafting did not differ from donor areas (Table 1).

**Table 1:** Mean and standard deviation (SD) for melanocyte percentages in skin biopsies from donor, receptor, grafted, pre-curettage, post-curettage vitiligo areas

Donor (mean % ± SD)	Receptor (mean % ± SD)	Grafted (mean % ± SD)	Pre-curettage (mean % ± SD)	Post-curettage (mean % ± SD)
3.68 ± 1.52	1.36 ± 0.83	2.90 ± 1.26	1.29 ± 0.70	1.81 ± 0.92

### Fontana-Masson silver stain for melanin

Five out of sixteen receptor areas (35%) showed melanin granules before grafting. Three of these five areas were longstanding vitiligo areas. For the remaining biopsy specimens, after curettage and/or grafting, the melanin distribution did not have a uniform pattern, and no relationship with the type of procedure was found.

### Tyrosinase mRNA by RT-PCR

**Figure 3:**
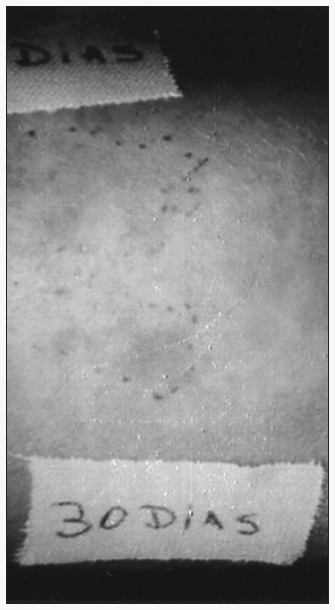
Polyacrylamide gel amplification of tyrosinase mRNA, detected via reverse transcriptase polymerase chain reaction (RT-PCR) 30 days after curettage of vitiligo.

All donor areas were positive for mRNA tyrosinase, as detected by means of RT-PCR. 93.75% of the vitiligo areas were negative for tyrosinase mRNA, including those that had positive Fontana-Masson staining for melanin. The receptor areas that initially were negative became positive for tyrosinase mRNA after grafting. Previously negative control areas became positive after curettage alone (84.2%) (Tables 2 to 4 and Figure 3).

**Table 2:** Tyrosinase mRNA (Ty-mRNA) detection results via reverse transcriptase polymerase chain reaction (RT-PCR), from vitiligo skin biopsies before grafting or curettage

	Positive Ty-mRNA	Negative Ty-mRNA	Total
Receptor area	1	11	12
Control area	1	19	20
**Total**	**2 (6.25%)**	**30 (93.75%)**	**32 (100%)**

**Table 3:** Tyrosinase mRNA (Ty-mRNA) detection results via reverse transcriptase polymerase chain reaction (RT-PCR), from receptor skin biopsies before and after grafting (among 12 receptor areas)

	Positive Ty-mRNA	Negative Ty-mRNA
Before grafting	1	11
After grafting	12 (100.0%)	None

*Conversion percentage = 100.0%.*

**Table 4:** Tyrosinase mRNA (Ty-mRNA) detection results via reverse transcriptase polymerase chain reaction (RT-PCR), from receptor skin biopsies before and after curettage for control areas (among 20 curetted areas)

	Positive Ty-mRNA	Negative Ty-mRNA
Before grafting	1	19
After grafting	17 (85.0%)	3 (15.0%)

*Conversion percentage = 84.2%.*

## DISCUSSION

Differences in the diffusion of melanin pigment from the graft to the adjacent areas give rise to variations in punch biopsy results between transplantation techniques.^21^ No matter how closely together tissue is grafted, pigmentation recovery tends to be uneven. Other techniques such as melanocyte culture have been proposed, but there is still much to be done to reach ideal results. Skin grafts prepared as explained above have resulted in homogeneous pigmentation, and have proven to be a valuable tool for longstanding localized vitiligo. Statistical analysis has shown that this finding is probably due to the addition of melanocytes, which reach the same levels as in the donor tissue. Furthermore, the number of melanocytes in these grafted areas exceed the proliferation induced by simple curettage.

A positive test for tyrosinase mRNA, by means of RT-PCR, was used as an indication of melanocyte activity, in all donor areas. Higher melanocyte percentages and tyrosinase positivity 30 days after grafting are indications that transplanted melanocytes are metabolically active. This may contradict the evidence put forward by Naeyaert^27^ (1991) that melanocyte activity is not related to the tyrosinase mRNA level, but regulated by post- transcriptional factors.

Five out of sixteen (31.25%) tyrosinase mRNA-negative vitiligo lesions showed silver stain for melanin in basal cell layer kerati- nocytes before grafting. The interpretation of this finding could be that it is the result of residual melanin pigmentation within vitiligo lesions. It could alternatively be that occasionally dormant melanocyte could have cyclical metabolic activity, thereby resulting in the transfer of melanosome into keratino- cytes. However, it should be borne in mind that the silver stain for melanin is not specific, and a false-positive reaction cannot be ruled out, although a positive control was stained together with the study samples. In findings favoring the possibility of residual pigmentation, Bartosik et al.^5^ (1998) reported that electron microscopy and histochemical reactions failed to identify melanocytes in older vitiligo lesions, albeit finding melanosomes within keratinocytes. Epidermal turnover is high, and we believe that it is very unlikely that pigment could be held in keratinocytes in longstanding lesions. For these reasons, we believe that dormant melanocytes are occasionally transformed into an active metabolic state under certain stimuli.

It may be possible to explain the partial perifollicular pigmentation, and the changing of 84.2% of vitiligo from negative to positive for tyrosinase mRNA after curettage alone, by the activation and proliferation of hair follicle bulb melanocytes or inducement of dormant melanocytes found in the initial anagen phase of the normal hair follicle cycle.^8^ This is probably another melanocyte pigment source for grafted vitiligo. There is still no explanation why simple curettage has an influence on the proliferation and metabolic activity of melanocytes in areas that are almost completely devoid of melanocytes. Repair of wounded tissue is influenced by stimulating factors from connective tissue and inflammatory cells, and these could possibly activate the melanocyte metabolism. Serum factors could also be part of this activation mechanism. In 1995, Abdel-Malek et al.^28^ reported that epidermal trauma induces keratinocytes to produce basic fibroblastic growth factor, which activates melanocyte membrane receptors for tyrosinase KIT-Kinase and, thereafter, mRNA nuclear transcription in dormant melanocyte. It is also well known that UVB radiation can induce keratinocytes to synthesize cytokines such as interleukin-1, tumor necrosis factor-alpha (TNF-alpha), transforming growth factoralpha (TGF-alpha) and nerve growth factor, and also arachidonic acid and its metabolites and all the stimulating factors and cofactors of fibroblast proliferation that are possibly related to melanocyte metabolic turnover.^29,30^

It may be possible to explain pigmentation that occurs in untreated lesions, far from the experimental areas, by systemic or wound-produced stimulating factors. This is an interesting finding that deserves further investigation.

## CONCLUSIONS

The homogeneous pigmentation, recovery to normal melanocyte percentage in the epidermal basal layer, and shift to positive tyrosinase mRNA state, are all favorable points regarding the efficacy of the proposed graft technique for longstanding vitiligo lesions. Simple curettage-induced partial pigmentation of vitiligo areas turned areas that had been negative for tyrosinase mRNA into areas that were positive, and increased the melanocyte percentage to levels that were greater than for the untreated vitiligo areas, although to a lesser degree than in the grafted areas. Our results suggest that dormant melanocytes within vitiligo lesions may be stimulated metabolically and induced to proliferate by endogenous and/or exogenous stimuli. However, further studies are necessary to clarify this issue.
